# The International Genome Sample Resource (IGSR) collection of open human genomic variation resources

**DOI:** 10.1093/nar/gkz836

**Published:** 2019-10-04

**Authors:** Susan Fairley, Ernesto Lowy-Gallego, Emily Perry, Paul Flicek

**Affiliations:** European Molecular Biology Laboratory, European Bioinformatics Institute, Wellcome Genome Campus, Hinxton, Cambridge CB10 1SD, UK

## Abstract

To sustain and develop the largest fully open human genomic resources the International Genome Sample Resource (IGSR) (https://www.internationalgenome.org) was established. It is built on the foundation of the 1000 Genomes Project, which created the largest openly accessible catalogue of human genomic variation developed from samples spanning five continents. IGSR (i) maintains access to 1000 Genomes Project resources, (ii) updates 1000 Genomes Project resources to the GRCh38 human reference assembly, (iii) adds new data generated on 1000 Genomes Project cell lines, (iv) shares data from samples with a similarly open consent to increase the number of samples and populations represented in the resources and (v) provides support to users of these resources. Among recent updates are the release of variation calls from 1000 Genomes Project data calculated directly on GRCh38 and the addition of high coverage sequence data for the 2504 samples in the 1000 Genomes Project phase three panel. The data portal, which facilitates web-based exploration of the IGSR resources, has been updated to include samples which were not part of the 1000 Genomes Project and now presents a unified view of data and samples across almost 5000 samples from multiple studies. All data is fully open and publicly accessible.

## INTRODUCTION

The International Genome Sample Resource (IGSR) maintains and expands the heavily used data resources created by the 1000 Genomes Project (1–4). The data provides a reference for background human genomic variation, based on samples contributed by individuals who were over 18 years of age and self-declared healthy. Uniquely, the 1000 Genomes Project data set encompasses 26 populations from across five continents, with ∼100 unrelated individuals from each population being present in the final panel, providing valuable information on allele frequencies. The sample collection also includes trios from each population. All samples have an unusually open consent, enabling all data to be fully publicly accessible and shared freely (https://www.internationalgenome.org/sample_collection_principles), allowing anyone to use the data.

The 1000 Genomes Project data has many uses, which include imputation, screening in the identification of pathogenic variants, supporting evolutionary and population genomics research, assessing the impact of variations on gene expression and more (5). The availability of cell lines further increases the utility of the resources, by enabling follow up on findings from the dataset and novel research building on the 1000 Genomes Project's results. This, combined with the existing wealth of data, means that the 1000 Genomes Project samples are also of particular use to those investigating new technologies and methods, where existing resources can be used for comparison. We continue to see an increase in the range of technologies applied to the samples, as exemplified by the work of the Human Genome Structural Variation Consortium (HGSVC) (6).

We provide a central integration point across several data generation projects for sequence and analysis results from 1000 Genomes Project samples and other samples with similarly open consent. Our resources, including links to data hosted on our FTP site or in the public archives, facilitate data discovery while avoiding duplication of elements in the archives. Our work includes collaboration with ongoing data generation projects to enable pre-publication data sharing. Available data include sequence data, alignments and variant calls. Formats vary with technology with, for example, specialized formats being used for optical maps. IGSR also shares intermediate ‘working’ files created during analysis. For all data, IGSR follows the principles of FAIR data sharing: Findable, Accessible, Interoperable and Reusable. IGSR ensures that data is in standard file formats, has sufficient metadata to enable users to understand what the data is and to make it easy for users to locate data sets of interest. This last aim drove the creation of the website's data portal, which has been developed further and enables exploration of the principal data sets in IGSR.

As IGSR grows, the 1000 Genomes Project data has been joined by additional data sets, which sit alongside the original resources. Among recent additions are outputs from reanalyzing the 1000 Genomes Project's data on the GRCh38 assembly, making the valuable 1000 Genomes Project resources available natively on the current human genome reference assembly. IGSR has realigned the sequence data from the 1000 Genomes Project to GRCh38 (7) and used those alignments to call variants (manuscript submitted, https://doi.org/10.12688/wellcomeopenres.15126.1). The range of data types available for samples continues to grow through ongoing collaboration with the HGSVC and the recent addition of high coverage sequence data for 2504 1000 Genomes Project samples from the New York Genome Center (NYGC), funded by NHGRI. Further expansion comes from the addition of populations from the Gambian Genome Variation Project (GGVP), the Simons Genome Diversity Project (SGDP) and the Human Genome Diversity Project (HGDP). The updated data portal (https://www.internationalgenome.org/data-portal) makes it possible to browse almost 5000 samples in a unified manner, including samples not present in the 1000 Genomes Project datasets.

## THE 1000 GENOMES SAMPLES ON GRCh38

Phase three of the 1000 Genomes Project used the GRCh37 reference assembly based on its availability at the start of analysis, meaning its resources were not generated on the improved GRCh38 assembly (8). Such resources are, however, required to fully utilize the newer assembly. While liftovers of data, using assembly mappings, have been created, these rely on being able to map successfully between the assemblies, which is not possible for all genomic regions. In addition, they do not replicate the outputs of reanalyzing the data *de novo* on the improved assembly. There is now, however, an increasing range of data from the 1000 Genomes Project samples that has been analysed directly on GRCh38 with many of the resulting resources available in IGSR.

### 1000 Genomes Project data updated to GRCh38

Sequence data produced by the 1000 Genomes Project was realigned to the GRCh38 assembly (7). This comprised low coverage WGS and exome data for 2548 unrelated samples and an additional 150 related samples. The alignments have been used in calling variants on GRCh38, in a process that used BCFtools, GATK and FreeBayes for site discovery and produced a joint-genotyped, integrated, phased, biallelic SNV call set in late 2018 (manuscript submitted, https://doi.org/10.12688/wellcomeopenres.15126.1). Since then, an updated call set, adding INDELs, has been released. All data from this work is available on the IGSR FTP site and all variants have been submitted to the European Variation Archive (EVA).

### High coverage data

In 2019, the New York Genome Center (NYGC), funded by NHGRI, released high coverage genomic sequence data for the 2504 unrelated samples in the 1000 Genomes Project phase three panel. Alignments of this data to GRCh38 and a GATK call set, both generated by NYGC, are currently accessible via the IGSR FTP site. This data set includes gVCFs and annotated calls. In addition, IGSR is sharing pre-publication call sets generated by groups using this data for variant detection, in particular those investigating structural variation.

### Multiple technologies

The breadth of technologies available on the GRCh38 assembly has continued to grow. This has been driven through collaboration with the HGSVC, which has focused on three trios from the 1000 Genomes Project sample collection. Technologies that have been applied to these samples include BioNano, Oxford Nanopore Technologies, 10X Genomics, Strand-seq and PacBio. Alignments and analyses of this data on GRCh38 were shared pre-publication via IGSR, with further details in the recent publication by Chaisson *et al.* (6). We anticipate that, through continued work with the HGSVC, the number of samples in the 1000 Genomes Project collection for which technologies such as Oxford Nanopore and PacBio are available will grow with ongoing pre-publication data sharing.

## INTEGRATING ADDITIONAL SAMPLES

Previously, we reported the addition of samples and data from the GGVP, the HGDP and the SGDP to our resources, with these data sets available via our FTP site (9). Since then, further work has been done to integrate these samples more fully and present them in the website's data portal, alongside the 1000 Genomes Project samples. In the case of the GGVP, samples were originally collected with the intention that they be part of the 1000 Genomes Project, meaning that the sampling strategy and metadata, including population definitions, are in harmony with samples from the 1000 Genomes Project. For the HGDP and the SGDP, however, the granularity with which populations are defined and the numbers of samples obtained from a given population vary significantly from the 1000 Genomes Project. Further, the inclusion of these samples takes the total number of populations represented in IGSR from 26 to over 200 populations. Additional complexity is introduced by the presence of certain samples in more than one project, with the same sample being assigned to different populations in different projects. An example of this is HG00128, which belongs to the ‘British from England and Scotland’ population and the European Ancestry superpopulation in the 1000 Genomes Project. The extended description for this population in the 1000 Genomes Project mentions that this population is comprised of samples from ‘Cornwall and Kent (England) and Orkney and Argyll & Bute (Scotland)’ (https://www.coriell.org/1/NHGRI/Collections/1000-Genomes-Collections/British-from-England-and-Scotland-UK-GBR). In SGDP, however, HG00128 is assigned to the English population and West Eurasia superpopulation, with the English population, from the description, a subset of the 1000 Genomes Project British population. Indeed, the SGDP English population consists of only two samples, HG00128 and HG00126, both of which are present in the 1000 Genomes Project British population. In another case, there are potentially ‘similar’ populations in different projects where, without any samples being shared, descriptive information for the populations suggests some possible similarity or overlap of the populations being sampled. An example of this is the case of Orcadian samples. As in the description above, the 1000 Genomes Project British population includes samples from Orkney and, while the HGDP and the SGDP have Orcadian populations, they do not share any common samples with the 1000 Genomes Project British population.

**Figure 1.**
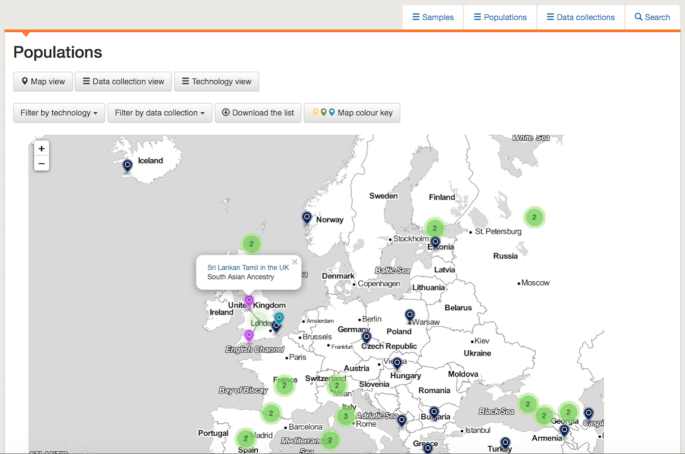
To support the increased number of populations in IGSR, a map view has been added to the website's data portal. This enables users to explore populations based on sampling location, with colouring indicating superpopulation groupings.

As can be seen in the 1000 Genomes Project publication (4) and the information provided by the Coriell Institute (https://www.coriell.org/1/NHGRI/Collections/1000-Genomes-Collections), significant consideration was given to the naming applied to the populations in the 1000 Genomes Project, with stipulations made as to what naming should be used. In order to meet the undertakings given by all of the original projects with respect to naming, we present samples in the population groupings in which they appear in contributing projects, seeking to leave the metadata from contributing projects intact. We do not attempt to combine samples into a single population scheme. Instead, we aim to present population, and superpopulation, information as clearly as possible, without assigning samples to new or different populations. Consequently, in IGSR, samples can belong to more than one population, and the corresponding superpopulations to which those populations belong. To support the increase in populations, the presentation of information in the data portal has been updated. A new map view, shown in Figure 1, plots populations by the sampling locations given by the contributing projects and colouring the map markers based on the superpopulation groupings to which the populations belong. Populations in this view can be filtered both by technologies applied to samples in the population and in which IGSR data collections the population can be found. The portal pages describing populations have been updated to list which, if any, samples are shared with other populations and sample level information now lists all populations to which a sample belongs.

## USING THE IGSR RESOURCES

### Exploring the data

Key data sets are added to the data portal (https://www.internationalgenome.org/data-portal) with all data available via the project FTP site (ftp://ftp.1000genomes.ebi.ac.uk), alongside the data sets created by the 1000 Genomes Project, which continue to be shared at this location. The data portal uses specific terminology to describe the data: *samples, populations, superpopulations, technologies* and *data collections*.

#### Samples

We use this term in relation to the original sample contributed by a donor. In this way, a sample refers to the original sample and material derived from it. This may differ from some resources, which can consider the product of a laboratory process to produce a distinct, new sample, with two samples and a relationship between those samples being recorded (10). There is a one-to-one mapping in IGSR between samples and donors.

#### Populations

These are defined by the projects from which samples have been added to IGSR, currently the 1000 Genomes Project, the GGVP, the HGDP and the SGDP. Samples can belong to more than one population and always belong to at least one population. Populations from the 1000 Genomes Project typically have around 100 samples. For other projects, populations are smaller; in some cases, there may be only a single sample.

#### Superpopulations

Populations are typically grouped into continental level groupings referred to as superpopulations. In the 1000 Genomes Project, the superpopulations reflect ancestry, rather than the current geographic location of the population. An example of this is the population ‘Indian Telugu in the UK’, which, while samples were collected in the UK, are in the South Asian Ancestry superpopulation. Superpopulations currently come from the 1000 Genomes Project, the HGDP and the SGDP. Each of these use different groupings.

#### Technologies

These are categories used to classify the data in IGSR based on the technologies and methods used in generating the data. Within a given technology category there may be some variation between data sets. Genomic sequence data described as ‘PCR-free high coverage’ might, for example, include data sets at 30× and 60× coverage. Technology categories aim to make it easier to identify data sets of a given general type but there may be variation between data inside that category.

#### Data collections

In IGSR data collections are used to identify a set, or collection, of data, typically associated with a given piece of research and its outputs. Data can belong to multiple collections. For example, there are collections for phase one and phase three of the 1000 Genomes Project (there was no defined phase two dataset), and another for reanalysis of the 1000 Genomes Project data on GRCh38. Some sequence data generated early in the 1000 Genomes Project appears in all three of these data collections because it was used in each of these analyses.

**Figure 2.**
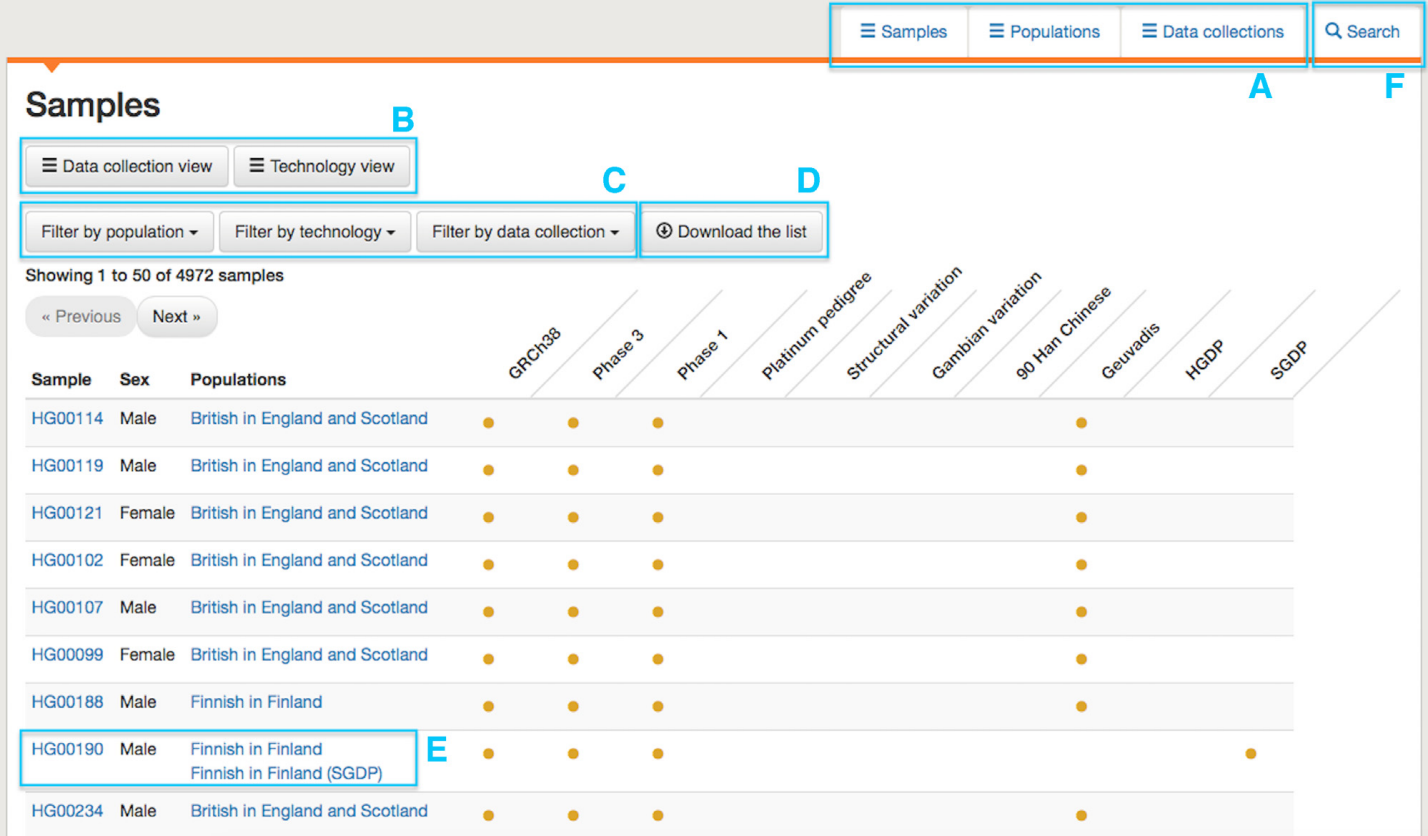
The data portal provides various views of key data sets in IGSR. (**A**) Data can be browsed by sample, population or data collection under different tabs. (**B**) On the sample and population tabs, different views of the data can be chosen. (**C**) Filters from multiple categories can be applied to the data. (**D**) The displayed data can be downloaded as a tab-delimited list. (**E**) In table views, information is displayed on the left, for samples, this is the sample name, sex and population information. This is followed by indicators, which, in data collection view, show which data collections the sample is present in. (**F**) The data portal also has search functionality, which can be accessed via the search tab or the search box present throughout the website.

**Figure 3.**
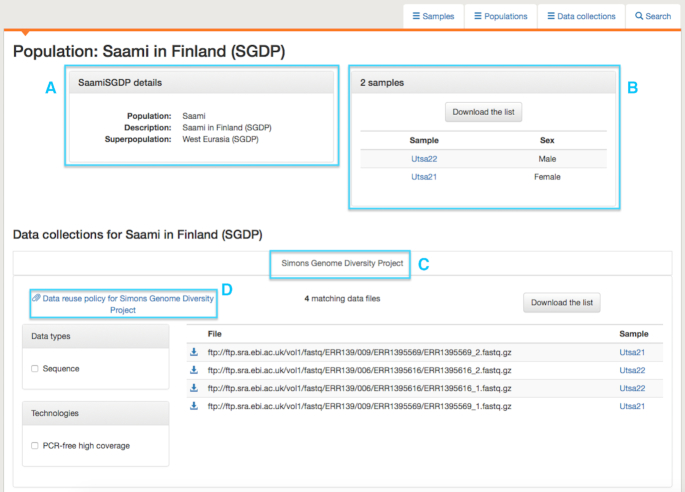
An example of a population page, showing the Saami population from the SGDP. (**A**) Gives some descriptive information. (**B**) Lists samples present in the population. (**C**) Tabs at the bottom of the page show which collections the population is found in, here only the SGDP. (**D**) For each collection, a link is provided to information on how the data may be used. If a population shares samples with another population, this information is presented on this page. In this case, no samples are shared.

**Figure 4.**
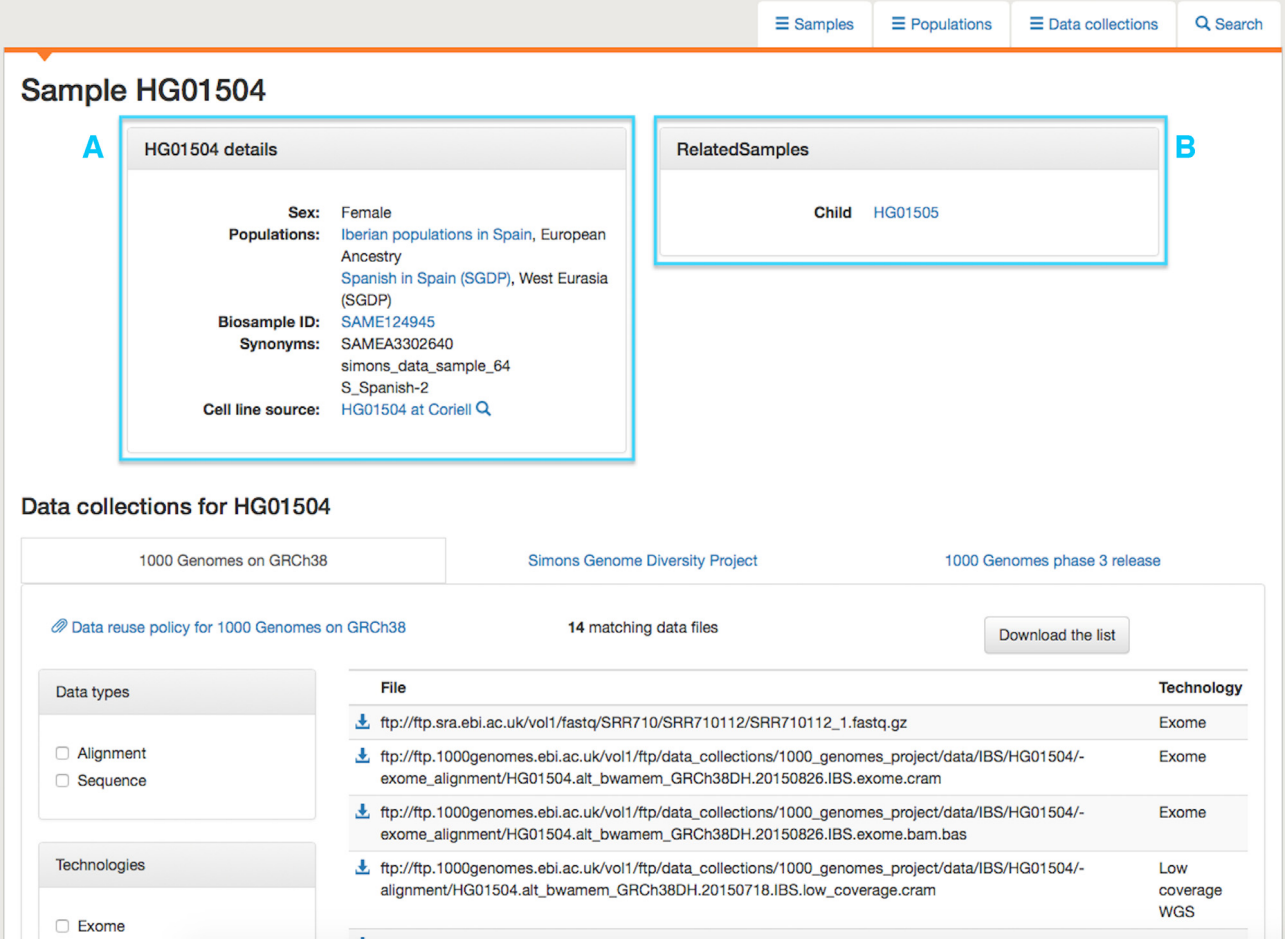
The page for sample HG01504. (**A**) Shows details on the sample. This sample belongs to populations in 1000 Genomes and the SGDP. Synonyms for the sample are also shown along with a link to the cell line source, where such exists, in this case Coriell. (**B**) Lists any known related samples present in IGSR.

In the data portal, information can be browsed by sample, population or data collection under different tabs, illustrated in Figure 2, which shows the sample tab and, in the selected view, indicates which data collections samples are present in. Alternatively, the view can be switched to display the technologies for which data exists for the sample. The list of samples can be filtered by population, technology or data collection, with the resulting list available to download as a tab delimited file. Under the population tab, views similar to those shown in Figure 2 are available, identifying which technologies and data collections a population is associated with, as well as the map view shown in Figure 1. Here, the populations shown can be filtered by technology and data collection and, again, a list can be downloaded. The data collection tab presents a list of the data collections currently available in the portal, along with summary information on how many samples and populations are present in the collection and links to any associated publications. The list of collections links to pages describing the collections in greater detail, including lists of populations and samples and, further down the page, a view of the files in the collection, which can be filtered using a set of checkboxes. Detailed information on populations and samples is also provided. An example of a population page is shown in Figure 3. This gives names and descriptions for the population, a list of samples, information on any overlapping populations (none are present in the example in Figure 3) and, at the bottom of the page, a set of tabs, one for each data collection that the population is represented in. For each collection, available files can be browsed and a link is provided to information on how the data may be used. Similar pages exist for each sample, shown in Figure 4. These include information on which populations a sample belongs to, any synonyms and any known related samples in IGSR. Where a cell line exists for the sample, a link is provided to the institute holding the cell line.

In addition to browsing in the portal a search facility is provided, highlighted in Figure 2. The search operates over the entire IGSR FTP site, not only the key data sets in the portal. Information on the site layout is available at ftp://ftp.1000genomes.ebi.ac.uk/vol1/ftp/README_ftp_site_structure.md, with additional information provided in adjacent README files. To mirror or track data on the FTP site, a change log is provided, with a summary at ftp://ftp.1000genomes.ebi.ac.uk/vol1/ftp/CHANGELOG.

### Accessing the data

#### Data reuse statements

Reuse policies vary between data collections in IGSR and data is frequently shared pre-publication. Users of the resources should consult the provided data reuse statements prior to using the data. Data reuse statements for collections are linked to from the portal and provided for each data collection on the FTP site. Our helpdesk will provide clarification if needed.

#### Data download

The majority of data in IGSR’s resources is hosted on either the project FTP site or the European Nucleotide Archive (ENA) FTP site. Details of download procedures vary slightly depending on where the data is hosted. Recommended methods for downloading data from IGSR are Aspera and Globus. Information on using these methods is provided on the project website.

### Browsing variation in Ensembl

IGSR primarily hosts data files and seeks to aid accessing files or subsets of the data that they contain. To view variation in a genomic context and with current annotation, we collaborate with the Ensembl Genome Browser (11). Ensembl makes the variation datasets from the 1000 Genomes Project available, provides tools to access sections of the data files and convert formats, and maintains up to date annotation as part of its presentation of variation data from a breadth of projects, including those with more limited consent, such as gnomAD (12). To search the 1000 Genomes Project call set by gene or rsID, we recommend using Ensembl and provide links to Ensembl from our search page.

### User support

We continue to provide an email-based help desk at info@1000genomes.org. Users are welcome to contact this address with any questions they may have about the resources. In addition, support is provided to users through an extensive list of FAQs.

## FUTURE WORK

As data on the samples in IGSR continues to grow, we anticipate adding more data to the resources. Files associated with the high coverage data from NYGC, currently on the FTP site, will be added to the data portal. We will also add the FASTQ files for this data, which are at present being archived. New call sets based on the high coverage data will also become available. IGSR plans to extend the range of available calls on GRCh38 further through generating calls for the samples in the GGVP. Planned and archived data sets have been identified, which will be added. These include data generated by the ongoing work of the HGSVC, ATAC-seq data generated for the 1000 Genomes Project British samples (ENA accession PRJEB28318) and HGDP transcriptome data (GEO accession GSE54308). We plan updates to the project website, to better describe the project to users and streamline the process of locating data and information. Beyond this, we will continue to offer direct support to users wishing to work with data contained in the resources.
